# Supplementary material for: A host-directed virulence factor of Clostridium perfringens is modulated by gut commensal strains
Source: Gut Microbes. 2026 Oct 29;18(1):2748768. doi: 10.1080/19490976.2026.2748768 (PMC13733201; doi:10.1080/19490976.2026.2748768)
Supplement: Supplementary file 1 [file KGMI_A_2748768_SM9452.docx]

**Tables**

**Supplementary Table 1.** Strains used in this study.

| **Strain** | **Relevant genotype and description** | **Reference or source** |
| --- | --- | --- |
| **Gut microbiome strains** | | |
| *Phocaeicola vulgatus* | DSM 1447 | Maier Lab |
| *Bacteroides uniformis* | DSM 6597 | Maier Lab |
| *Bacteroides fragilis* | DSM 2151 | Maier Lab |
| *Bacteroides thetaiotaomicron* | DSM 2079 | Maier Lab |
| *Thomasclavelia ramosa* | DSM 1402 | Maier Lab |
| *Agathobacter rectalis* | DSM 17629 | Maier Lab |
| *Roseburia intestinalis* | DSM 14610 | Maier Lab |
| *Fusobacterium nucleatum* subsp*. Nulceatum* | DSM 15643 | Maier Lab |
| *Enterocloster bolteae* | DSM 15670 | Maier Lab |
| *Clostridium perfringens* C36 | Isolate from human feces | DSMZ, DSM 11782 |
| *Clostridium perfringens* C36 Δ*plc* | Clean *plc* deletion strain | This study |
| *Clostridium perfringens* C36 Δ*iolE* | Clean *iolE* deletion strain | This study |
| *Clostridium perfringens* C36 pMTL83151-Ppfo-sLuc | Strain harboring a reporter plasmid with the luciferase gene *sLuc* under the control of the perfringolysin O promoter | This study |
| *Lacrimispora saccharolytica* | DSM 2544 | Maier Lab |
| *Streptococcus salivarius* | DSM 20560 | Maier Lab |
| *Ruminococcus gnavus* | VPI C7-9, ATCC 29149 | Maier Lab |
| *Bariatricus comes* | VPI CI-38, ATCC 27758 | Maier Lab |
| *Parabacteroides merdae* | DSM 19495 | Maier Lab |
| *Streptococcus parasanguinis* | DSM 6778 | Maier Lab |
| *Collinsella aerofaciens* | DSM 3979 | Maier Lab |
| *Dorea formicigenerans* | DSM 3992 | Maier Lab |
| *Clostridium sporogenes* | Type strain, soil isolate | DSMZ, DSM 795 |
| *Clostridioides difficile* | Strain 630, clinical isolate | DSMZ, DSM 27543 |
| ***E. coli* strains** | | |
| *Escherichia coli* NEB5α | *fhuA2Δ(argF-lacZ)U169 phoA glnV44 Φ80Δ(lacZ)M15 gyrA96 recA1 relA1 endA1 thi-1 hsdR17* | New England Biolabs |
| *Escherichia coli* ST18 | Strain for di-parental mating, auxotrophic for 5-aminolevulinic acid | DSMZ, DSM 22974 |
| *Escherichia coli* ST18 pMTL83251-Δ*plc* | Strain harboring pMTL83251-Δ*plc* to introduce *plc* deletion in *C. perfringens* | This study |
| *Escherichia coli* ST18 pMTL83251-Δ*iolE* | Strain harboring pMTL83251-Δ*iolE* to introduce *iolE* deletion in *C. perfringens* | This study |
| *Escherichia coli* ST18 pMTL83251-P*_pfo_*-*sLuc* | Strain harboring the pMTL83251-P*_pfo_*-*sLuc* reporter plasmid | This study |

**Supplementary Table 2.** Species-specific primers used in this study.

| **Primer name** | **Sequence (5’ to 3’)** | **Source** |
| --- | --- | --- |
| *P. vulgatus* fwd | gcacggggcacaatctctatatctgta | This study |
| *P. vulgatus* rev | cggaaaacggagtggactacacaagtg | This study |
| *B. uniformis* fwd | cctgcgcacgcagttgttttatag | This study |
| *B. uniformis* rev | caagggaagtatatgacgtttcaggtg | This study |
| *B. fragilis* fwd | ggaactgatagcttggacacagga | This study |
| *B. fragilis* rev | ctgtctgaggaatcatcgttatacgtgta | This study |
| *B. thetaiotaomicron* fwd | gagcgactggatgtttcctctctgattg | This study |
| *B. thetaiotaomicron* rev | ccggcatcattacggagtattagccc | This study |
| *T. ramosa* fwd | cgttgaatcaatgagaggggcaaataac | This study |
| *T. ramosa* rev | ctcatcaaattgctctggaccaatcatac | This study |
| *A. rectalis* fwd | gtgggcgagctcgtttattcctgag | This study |
| *A. rectalis* rev | cctgagcatatttccaaacaccatgatacc | This study |
| *R. intestinalis* fwd | caatcactcagacagtagcaaagccag | This study |
| *R. intestinalis* rev | gttgtcagctttgcgagtttcttcgatg | This study |
| *F. nucleatum* fwd | gtgaaagatttgattgggaaatagcaac | This study |
| *F. nucleatum* rev | cttaaatgctgcaatacctcctgtaactcc | This study |
| *E. bolteae* fwd | gatactggatgtggagggactggggg | This study |
| *E. bolteae* rev | cggaatatagacatccttcctcaggcc | This study |
| *C. perfringens* fwd | gccattataagtttggacttaggtaaagggc | This study |
| *C. perfringens* rev | ctctgcaaactttctcccctttgaatC | This study |
| *L. saccharolytica* fwd | ggtaaatccagaccagatatattccggc | This study |
| *L. saccharolytica* rev | cagcatcctggaaatcctcttttttgaag | This study |
| *S. salivarius* fwd | gcagaacgagaaatggagctatcacaag | This study |
| *S. salivarius* rev | catacaagaaactagaaccatctccgtctc | This study |
| *R. gnavus* fwd | ggattttgtcgtatctcctgtgaaattcgg | This study |
| *R. gnavus* rev | ccgctttattatgcgcagatgaatggaac | This study |
| *B. comes* fwd | gtcatagaaaccggtaaaatcaacctgcgc | This study |
| *B. comes* rev | ccgaaattgcctttcaaatgcggatattcc | This study |
| *P. merdae* fwd | ccaggaggattatctgcctatatagggg | This study |
| *P. merdae* rev | gggtaatcagaagtccaaaaatgagtccc | This study |
| *S. parasanguinis* fwd | gcgtattgttgaacgtgtagagtggttg | This study |
| *S. parasanguinis* rev | gaaggagagggccagtgtattatagatcac | This study |
| *C. aerofaciens* fwd | ctaagtactatggccgctttaaccaggg | This study |
| *C. aerofaciens* rev | ccacgagcagtttgtcgatcgtctc | This study |
| *D. formicigenerans* fwd | ccggtacttgctcttccgaatcttatattc | This study |
| *D. formicigenerans* rev | gtcgtataggaaaaacgcaacaagccc | This study |
| *C. sporogenes* fwd | ggactaatttgtccaggccatgtagcttc | This study |
| *C. sporogenes* rev | cctaagccagagtctttaattaatcctatgcc | This study |
| *C. difficile* fwd | gttattttaggcgctgagttggcacag | This study |
| *C. difficile* rev | ctgtgcattttgagcaaagaaacctacttg | This study |
| 515F | gtgccagcmgccgcggtaa | Caporaso *et al.* (2011) |
| 806R | ggactachvgggtwtctaat | Caporaso *et al.* (2011) |

**Supplementary Table 3.** Primers used in this study for genetic modification.

| **Primer name** | **Sequence (5’ to 3’)** | **Source** | **Function** |
| --- | --- | --- | --- |
| M13-fwd | tgtaaaacgacggccagt | Eurofins genomics (Konstanz, Germany) standard primer list | Sequencing |
| M13-rev | caggaaacagctatgacc | Eurofins genomics (Konstanz, Germany) standard primer list | Sequencing |
| seq RiboCas-f | ctgtatttcccagaaccttgaac | This study | Sequencing |
| seq RiboCas-r | gctatagatagagttctctaacggc | This study | Sequencing |
| Dplc-screen-f | catgttctcctaataagagtgc | This study | Sequencing |
| Dplc-screen-r | ccctagttaatattactgtgtttgc | This study | Sequencing |
| iolE_seq_fwd | ggtaaaataggaaatccacac | This study | Sequencing |
| iolE_seq_rev | cctaatataggtactaaaagacc | This study | Sequencing |
| gRNA+term f2 | gttttagagctagaaatagcaagttaaaataaggctagtc  cgttatcaacttgaaaaagtggcaccgagtcggtgcataaaa | This study | Cloning |
| gRNA+term r2 | gacgtcataaaaataagaagcctgcaaatgcaggcttcttattt  atcaacttgaaaaagtggcaccgagtcggtgcataaaa | This study | Cloning |
| plc-RHA-f | cgggggatataaaaataaaagtaaaaaataattattggttttgg | This study | Cloning |
| plc-RHA-r | taatggcggcgcgcctctataaaaatcccc | This study | Cloning |
| plc-LHA-f | tatttttatgacgtcaaatgaccaaattgg | This study | Cloning |
| plc-LHA-r | tttacttttatttttatatcccccgttaatttttttc | This study | Cloning |
| CperfPfdx-fwd | tgaccatgattacgaattcgagctcggtacactagaatagg  ctaaatatgcttaaaagag | This study | Cloning |
| Pfdx-plc-rev | gctctaaaactgcattaaaatctttgttttaataaaacacctc  ctaaaatgttggtataa | This study | Cloning |
| Pfdx-iolE-rev | gctctaaaacgaatcaaataataagtaaacaataaaacacc  tcctaaaatgttggtataa | This study | Cloning |
| gRNA-iolE-fwd | gtttacttattatttgattcgttttagagctagaaatagcaag | This study | Cloning |
| LHA-iolE-fwd | cttcttatttttatgacgtccaattcatgattttgatatggctgc | This study | Cloning |
| LHA-iolE-rev | cattattgaccaaatttatctctattcccccattacctttg | This study | Cloning |
| RHA-iolE-fwd | gggggaatagagataaatttggtcaataatgaattttagg | This study | Cloning |
| RHA-iolE-rev | caggcctcgagatctccatggacctactacccctaataaaaatcc | This study | Cloning |
| Ppfo-sLuc-fwd | agctatgaccgcggccgctgtatccatagtggattcaaattccat  aaatggaac | This study | Cloning |
| LHA-pfo-rev | aattttttacttggtctcataatataaatccccctttttt | This study | Cloning |
| sLuc-pfo-fwd | gatttatattatgagaccaagtaaaaaattattaatag | This study | Cloning |
| Ppfo-sLuc-rev | catgtctgcaggcctcgagatctccatgctatgctagaata  cgttcacaaagtc | This study | Cloning |

**Supplementary Table 4. Plasmids used in this study.**

| **Plasmid** | **Description** | **Reference or source** |
| --- | --- | --- |
| pMTL83151 | ColE1 + *traJ*, pCB102, and *catP* | Heap, J. T. et al., 2009 |
| pMTL83251 | ColE1 + *traJ*, pCB102, and *ermB* | Heap, J. T., et al., 2009^83^ |
| pFF189 | *catP*, P*_fdxA_*-sLuc (the sLuc gene is codon-optimized for *C. difficile*) | Schumacher *et al.* (2025) |
| pRECas1 | ColE1 + *traJ*, pCB102, *catP*, P*_fdx_*-riboswitch E-*cas9*, and P*_araE_* | Cañadas *et al.* (2019) |
| pRECas2 | ColE1 + *traJ*, pCB102, *catP*, P*_fdx_*-riboswitch E-*cas9*, and P*_J23119_* | Cañadas *et al.* (2019) |
| pRGCas1 | ColE1 + *traJ*, pCB102, *catP*, P*_fdx_*-riboswitch G-*cas9*, and P*_araE_* | Cañadas *et al.* (2019) |
| pRGCas2 | ColE1 + *traJ*, pCB102, *catP*, P*_fdx_*-riboswitch G-*cas9*, and P*_J23119_* | Cañadas *et al.* (2019) |
| pMTL83251-Δ*plc* | ColE1 + *traJ*, pCB102, *ermB*, gRNA scaffold and target region in the *plc* gene, 600bp upstream region of *plc*, and 600bp downstream region of *plc* | This study |
| pMTL83251-Δ*iolE* | ColE1 + *traJ*, pCB102, *ermB*, gRNA scaffold and target region in the *iolE* gene, 600bp upstream region of *iolE*, and 600bp downstream region of *iolE* | This study |
| pMTL83151-P*_pfo_*-*sLuc* | ColE1 + *traJ*, pCB102, *catP*, and P*_pfo_*-*sLuc* (*sLuc* codon-optimized for *C. difficile*) | This study |

**Supplementary Table 5.** SIRIUS Compute settings

| ***SIRIUS - Molecular Formula Identification*** | |
| --- | --- |
| General | |
| Instrument | Orbitrap |
| MS2 mass accuracy (ppm) | 5 |
| Fix formula for detected lipid | yes |
| Fallback Adducts | [M + H]+, [M + K]+, [M + Na]+ |
| Molecular formula generation | De novo + bottom up (recommended) |
| Perform de novo below m/z | 400 |
| Apply element filter to | De novo |
| Allowed elements | H, C, N, O, P |
| ***ZODIAC - Network-based improvement of SIRIUS molecular formula ranking*** | |
| OFF |  |
| ***Predict properties: CSI:FingerID - Fingerprint Prediction & CANOPUS - Compound Class Prediction*** | |
| ON |  |
| Score threshold | yes |
| ***CSI:FingerID - Structure Database Search*** | |
| ON |  |
| Search strategy | |
| PubCham as fallback | yes |
| Confidence mode | APPROXIMATE |
| Search DBs | Biocyc, Blood Exposome, CHEBI, COCONUT |
|  | |
| **Note:** compounds with mass higher than 850 Da were excluded as fragmentation tree computation takes too long | |

**Supplementary Figure 1: Com18 composition and growth of monocultures and co-cultures. A:** Phylogenetic tree of Com18 including *C. perfringens* and the two other pathobionts, *C. difficile* and *C. sporogenes*. The phylogenetic tree was generated using iTOL^84^. **B:** Relative abundance of each strain of Com18 (and *C. perfringens*) in the absence (top, “- *Clostridium perfringens*”) or presence (bottom, “+ *Clostridium perfringens*”) of *C. perfringens*. R1-3: bioreactor replicates without *C. perfringens*, R4-6: bioreactor replicates with *C. perfringens*, the triplicates are represented in one panel per timepoint. The first two time points originate from batch operation, and the following three time points from continuous operation. The relative abundances were determined *via* 16S rRNA sequencing. **C:** Growth curves of all strains in single cultures over 24 hours. The standard deviations are shown as areas adjacent to the mean growth (N = 3). **D:** The relative growth of *C. perfringens* in co-culture plotted against the relative growth of the community strains in co-culture. The grey line represents the linear trend line. The Spearman coefficient (ρ) and p-value are indicated in the top right. Each co-culture was colored based on the cluster into which the community strain falls as shown in **Figure 2A**, and the shape represents the interaction type.

**Supplementary Figure 2: Competition for amino-acids in co-cultures. A:** Heatmap of proteins that were found to be more abundant in the co-cultures compared to the single culture. Each protein was classified according to the COG database and the number of proteins in each class is visualized in the heatmap. The strains are arranged according to the Euclidean distance metric. Mean values of triplicates were used. **B:** Amino acid biosynthesis pathway as shown by KEGG^85^ (map01230). *C. perfringens* enzymes annotated in the genome are shown as black arrows, and grey arrows symbolize absent genes. Amino acids in filled red boxes were shown to be essential in several studies. Amino acids with a red border were found to be essential in at least one publication^6,7,86^. **C:** Abundance of the *B. fragilis* protein Q5LEZ7 in the *B. fragilis* monoculture and in the *C. perfringens* co-culture (co-culture).

**Supplementary Figure 3: Proteome response of *C. perfringens* in co-cultures. A:** Heatmap of *C. perfringens* proteins that were found to be less abundant or absent in the co-cultures compared to the single culture. **B:** Heatmap of *C. perfringens* proteins that were found to be more abundant or present in the co-cultures compared to the single culture. **A and B:** Each protein was classified according to the COG database and the number of proteins in each class is visualized in the heatmap. Euclidean distance metric was used to determine the relationship. Mean values of triplicates were used. **C:** UpSet plot of *C. perfringens* proteins that are present or more abundant in the co-culture than the single culture. Only proteins that were found in at least two cultures are included.

**Supplementary Figure 4: Metabolite classes of the cell-associated metabolomes.** Pie charts with metabolite classes found in the metabolomes of *P. vulgatus*, *B. uniformis*, *B. fragilis*, *B. thetaiotaomicron*, and *C. perfringens.*

**Supplementary Figure 5: Metabolite classes of the exometabolomes.** Pie charts with metabolite classes found in the metabolomes of *P. vulgatus*, *B. uniformis*, *B. fragilis*, *B. thetaiotaomicron*, and *C. perfringens*.

**Supplementary Figure 6: Sphingolipids in Com18 monocultures and co-cultures.** Sphingolipids detected *via* FI-MS in all single- and co-cultures grouped by family. *C. perfringens* is shown separately from the *Clostridiaceae*. The intensity was normalized to the mean intensity of each metabolite across all samples. All replicates are shown in the heatmap (N = 3).

References

6. Goldner SB, Solberg M, Post LS. Development of a minimal medium for *Clostridium perfringens* by using an anaerobic chemostat. ApEnM. 1985;50:202–206. doi: 10.1128/aem.50.2.202-206.1985.
7. Fuchs A-R, Bonde GJ. The nutritional requirements of *Clostridium perfringens*. Microbiology. 1957;16:317–329. doi: 10.1099/00221287-16-2-317.
83. Rossjohn J, Parker M, Polekhina G, Feil S, Tweten R. in Worldwide Protein Data Bank. 2004.
84. Letunic I, Bork P. Interactive tree of life (iTOL) v6: recent updates to the phylogenetic tree display and annotation tool. Nucleic Acids Res. 2024;52:W78–W82. doi: 10.1093/nar/gkae268.
85. Kanehisa M, Furumichi M, Sato Y, Kawashima M, Ishiguro-Watanabe M. KEGG for taxonomy-based analysis of pathways and genomes. Nucleic Acids Res. 2023;51:D587–D592. doi: 10.1093/nar/gkac963.
86. Sebald M, Costilow RN. Minimal growth requirements for *Clostridium perfringens* and isolation of auxotrophic mutants. Appl Microbiol. 1975;29:1–6. doi: 10.1128/am.29.1.1-6.1975.
